# The relationship between menstrual cycle pattern and post‐traumatic stress in women following the 2023 earthquake in Turkey

**DOI:** 10.1002/brb3.70034

**Published:** 2024-09-25

**Authors:** Sibel Kiyak, Serap Batı

**Affiliations:** ^1^ Seydişehir Kamil Akkanat Faculty of Health Sciences, Department of Nursing Necmettin Erbakan University Konya Turkey

**Keywords:** earthquake, menstrual cycle, post‐traumatic stress disorder

## Abstract

**Aim:**

In the aftermath of natural disasters, understanding the intricate links between mental health and physiological responses, such as menstrual cycle patterns, becomes crucial. This study explores the relationship between post‐traumatic stress and menstrual irregularities among women of reproductive age residing in regions affected by the 2023 earthquake in Turkey.

**Methods:**

309 women of reproductive age living in 11 provinces affected by the earthquake centered in Kahramanmaraş on February 6, 2023 and declared as disaster areas constituted the study sample. Data were collected online using Google forms nine months after the earthquake. The collected data were obtained using the Participant Information Form, Impact of Events Scale and Post‐traumatic Stress Disorder‐Short Form. In the data analysis, descriptive statistics such as count, percentage, median, minimum, and maximum were used, along with statistical tests including the Mann–Whitney *U* test, chi‐squared test, multiple logistic regression analysis, and ROC analysis.

**Results:**

In the study, an increase in menstrual irregularities among women was observed following the earthquake (%14.3 to %44.8, *p* < .001). Risk factors for menstrual irregularities included post‐traumatic stress symptoms, comorbid chronic diseases, and smoking. The prevalence of PTSD was found to be 22.7% and this was associated with women with menstrual irregularities. Setting the cut‐off score of the IES‐R scale at 45.50 resulted in higher sensitivity for detecting irregular menstrual cycles.

**Conclusions:**

Women's menstrual cycles are affected after an earthquake. Therefore, post‐earthquake mental health recovery programs should specifically address the protection of women's physical and mental health. This comprehensive approach can reduce the effects of earthquake‐induced stress and trauma.

## Graphical abstract

Nine months after the disaster, menstrual irregularities increased from 14.3% to 44.8%, and PTSD prevalence was 22.7%. Risk factors for menstrual irregularities included post‐traumatic stress symptoms, comorbid chronic illnesses, and smoking.

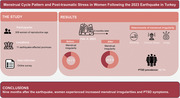

## 1 LIMITATIONS

This study has certain limitations. Data were collected 9 months after the 2023 Kahramanmaraş earthquake sequence using an online self‐report method. The online collection of data allowed the participation of only women with Internet access. Therefore, the study participants may not be fully representative of women in the earthquake‐affected zone. A causal relationship could not be established due to cross‐sectional descriptive and retrospective data. It should be taken into account that due to the time elapsed from the earthquake sequence to the interviews, items about the menstrual cycle pattern before the earthquake might have caused recall bias in the data.

## 2 INTRODUCTION

In 2023, Turkey was shaken by two consecutive major earthquakes, measuring M7.7 and M7.6 on the Richter scale, with epicenters at Pazarcık and Elbistan towns of Kahramanmaraş province, Southeastern Turkey. The Kahramanmaraş earthquake sequence resulted in nearly 50,783 deaths, 115,353 injuries, and the collapse of nearly 37,984 buildings (AFAD, 2023). In addition to the loss of life and physical damage, traumatic effects emerged as a result of aftershocks, loss of loved ones, and homelessness during the aftermath of the earthquakes (Aurizki et al., 2019). The loss of beloved individuals, homelessness, and displacement in the aftermath of the earthquake was associated with an increased risk of psychological problems for the survivors (Wu et al., 2014). Menstrual cycle irregularity is one of the healthcare issues associated with the above traumatic effects.

Regular menstrual cycle is often considered a reflection of healthy body functioning. Furthermore, the menstrual cycle is defined as the regular course of events from menarche to menopause, producing an ovum ready for fertilization and endometrium ready for implantation each month (Berga, 2020). This process is characterized by predictable fluctuations in ovarian hormones, including estrogen and progesterone (Schmalenberger et al., 2021). A regular menstrual cycle requires a normally functioning hypothalamic–pituitary–gonadal (HPG) axis and hormonal feedback loops (Itriyeva, 2022). Stress activates the HPG axis, inducing the release of stress hormones such as cortisol. High cortisol levels are associated with the suppression of the HPG axis and decreased gonadotrophin‐releasing hormone activity, leading to decreased follicle‐stimulating hormone and luteinizing hormone levels and impaired ovarian function (Palm‐Fischbacher & Ehlert, 2014). Any change in the hormonal feedback loops may affect menstrual cycle characteristics, including cycle pattern, duration, frequency, and amount of bleeding (Fraser et al., 2011). These irregularities may occur in a range of forms, including dysmenorrhea, oligomenorrhea, polymenorrhea, abnormal vaginal bleeding, menorrhagia, and amenorrhea (Mahmood & Jabeen, 2013; Taşkın, 2019). The stress associated with earthquakes can induce changes in hormonal balance in women, which in turn causes irregular menstrual cycles. In a study following the Wenchuan earthquake in China, 21% of affected women reported menstrual irregularities (Li et al., 2011). Similarly, 42.4% of women reported menstrual irregularities in the aftermath of the 2017 Kermanshah earthquake in Iran (Rajabi et al., 2022). Therefore, stress in women as a result of traumatic events such as earthquakes is considered a factor that can drastically alter the regularity of the menstrual cycle and have an adverse effect on the reproductive health of affected women.

Post‐traumatic stress disorder (PTSD) is a psychiatric condition characterized by symptoms of fear, panic, helplessness, avoidance, living the traumatic event over, and hyperarousal, which may occur after the affected person has been exposed to a serious traumatic event (American Psychiatric Association & Association, 2013). It was reported that PTSD was the most prevalent mental health condition among earthquake survivors (Farooqui et al., 2017). Previous studies reported that 23.6%−40.1% of post‐earthquake survivors had PTSD (Dai et al., 2016; Wu, Xu, & Sui, 2016). Women, in particular, may be more likely to suffer from earthquake‐related PTSD (Mu et al., 2022; Zhou et al., 2015). Earthquakes are powerful stressors that can adversely impact women's menstrual cycle and reproductive health by affecting the pituitary gland through the cerebral cortex and hypothalamus (Liu et al., 2010). Studies with students affected by earthquakes reported that students with PTSD symptoms had higher rates of irregular menstrual cycles (Liu et al., 2010), premenstrual syndrome (Takeda et al., 2013a), and dysmenorrhea (Takeda et al., 2013b).

Menstrual cycle irregularities may form the basis of several medical conditions, including metabolic syndrome, coronary heart disease, anemia, type 2 diabetes, rheumatoid arthritis, and infertility (Attia et al., 2023). Genetic predisposition, dietary habits, obesity, physical activity, sleep patterns, smoking, environmental exposures, and stress may play a significant role in menstrual cycle irregularity (Aurizki et al., 2019; Bae et al., 2018). Nevertheless, the potential effects of post‐earthquake traumatic experiences on the menstrual cycle are often neglected. The severe stress and psychological effects caused by earthquakes can disrupt hormonal balance, leading to irregularities in the menstrual cycle. Understanding this relationship can help us grasp the impact of post‐disaster psychological effects on women's health in the long term. Accordingly, the present study aimed to fill a significant gap in the relevant literature by investigating the relationship between alterations in menstrual cycles and PTS earthquake‐affected women. Additionally, whether the post‐traumatic stress score can be used to distinguish between regular and irregular menstrual cycles was determined as a secondary aim.

### 2.1 Research questions

1. What is the prevalence of menstrual cycle irregularities in women in the aftermath of an earthquake?
2. What is the rate of PTS in women after an earthquake?
3. Is there an association between menstrual cycle pattern and PTS in women affected by an earthquake?
4. Can the post‐traumatic stress score be used to distinguish between regular and irregular menstrual cycles?

## 3 METHODS

### 3.1 Participants

This study, designed as descriptive and correlational research, was conducted online via Google Forms 9 months after the 2023 Kahramanmaraş earthquake sequence (November 6–30, 2023). The study covered 11 provinces, namely, Kahramanmaraş, Adana, Adıyaman, Diyarbakır, Gaziantep, Hatay, Kilis, Malatya, Osmaniye, Şanlıurfa, and Elazığ, which were affected by the Kahramanmaraş earthquake sequence on February 6, 2023, and included in the disaster zone declared upon the earthquake. Women of reproductive age (18−49 years) who were literate in Turkish and had Internet access were included in the study. Pregnant women, postpartum and/or lactating women, menopausal women, women diagnosed with polycystic ovary syndrome, endometriosis or hyperthyroidism, women who underwent gynecological operations, including hysterectomy, and women previously diagnosed with a psychiatric disorder (by self‐report) were excluded. The size of the study sample was set to 272 individuals and a confidence level of 95%, a test power of 95%, and an effect size of 0.202 (Kvestad et al., 2019) were considered.

### 3.2 Procedure

Relevant approval of the Ethics Committee of the Faculty of Health Sciences, Necmettin Erbakan University (Ethics Committee Approval No: 579, Date: 01.11.2023) was obtained prior to the commencement of the study. The study was conducted under the principles stipulated in the World Medical Association Declaration of Helsinki‐ethical principles for medical research involving human subjects. The study data were collected online using the snowball sampling method 9 months post‐earthquake. The questionnaire and consent form link were forwarded to the participants online. The consent form included information about the purpose and method of the study, which the participants could read before completing the questionnaire. The participants who responded affirmatively to the statement, “I was duly informed about the study. I agree to participate.” provided their responses to the questionnaire items.

### 3.3 Measures

#### 3.3.1 Participant Information Form

The questionnaire form, previously created by the researcher based on a literature review, consisted of 35 items on the socio‐demographic (age, education, working status, and marital status), general living habits (chronic illness, smoking, weekly physical activity, sleep duration, and family planning method use), earthquake‐related (place of residence during the earthquake, where to live after the earthquake, financial loss due to earthquake, loss of health due to earthquake, and staying in a tent/container after the earthquake), and menstrual characteristics (menarche age, current and pre‐earthquake menstrual duration, frequency, and bleeding intensity) of the participants.

#### 3.3.2 Irregular menstruation

Menstrual irregularity was assessed through questions regarding self‐reported menstrual problems in the survey. Participants were asked, “Is your menstrual cycle regular right now?” and “Was your menstrual cycle regular before the earthquake?” Those who answered “yes” were considered to have a regular menstrual cycle, while those who answered “no” had an irregular one. For the purposes of the study cycles, which lasted shorter than 21 days (polymenorrhea) and longer than 35 days (oligomenorrhea), bleeding at any time other than menstrual bleeding (metrorrhagia), vaginal bleeding, which required changing multiple tampons or pads in 1−2 h (menorrhagia), and cessation of menstruation for 3 months or longer (secondary amenorrhea) were considered as menstrual irregularities based on the reports of the participants (9). The severity of menstrual bleeding was classified by the participants themselves as mild, moderate, and severe.

#### 3.3.3 Post‐traumatic stress

The impact of event scale‐revised (IES‐R) was used to measure the stress level of people who were exposed to any trauma. The scale was originally developed by Horowitz and Wilner (Horowitz et al., 1979) and thereafter revised by Weiss and Marmar (Weiss & Marmar, 1997). The adaptation of the scale to the Turkish language was made by Çorapçıoğlu and Yargıç (Çorapçıoğlu et al., 2006). The 5‐point Likert‐type scale comprises 22 items and 3 sub‐domains (intrusion, avoidance, and hyperarousal). The 5‐choice responses are scored between 0 and 4; the lowest and highest total scores from the scale are 0 and 88, respectively. For the purposes of the study, the traumatic life event specified in the scale was defined as having been exposed to the earthquake. “Even such things that are unrelated and different from the earthquake remind me of the event, bring it to my mind, and make me think about it” is a sample item. The Cronbach's alpha coefficient was calculated as 0.91 in the study.

The Post‐Traumatic Stress Disorder‐Short Scale (PTSD) is a brief nine‐item self‐report scale that can be used to assess PTSD and measure its severity. It was originally developed by LeBeau and Mischel (LeBeau et al., 2014), and the Turkish validity and reliability study thereof was conducted by Evren and Dalbudak (Evren et al., 2016). Each item in the scale is scored between 0 and 4 points (not at all = 0, a little = 1, moderate = 2, quite = 3, and extreme = 4). The total score from the scale varies between 0 and 36, yet a score of 24 points and above is considered a significant level for PTSD. Furthermore, the traumatic life event specified in the scale was defined as exposure to the earthquake. The general description part of the scale was adapted as follows: “To what extent did each of the following problems that emerged or worsened after the earthquake bother you in the past 7 days?” In the study, the Cronbach's alpha coefficient was calculated as 0.87.

#### 3.3.4 Covariates

The study included several covariates with potential impact on menstrual cycle irregularity. Based on a review of previous studies, several likely confounding variables on menstrual cycle irregularity were considered, including age, marital status, and body mass index (BMI) (Alhammadi et al., 2022; Kwak et al., 2019). For the marital status, participants were categorized as married (currently living with a partner) and single (single, divorced, and widowed). BMI was calculated by dividing the body weight (kg) by the square of the height (m^2^).

### 3.4 Data analysis

The Statistical Package for the Social Sciences (IBM SPSS V29, Chicago, USA) software was used to analyze the study data. The Kolmogorov–Smirnov test was used to test the hypothesis of the normal distribution of the values by groups. The Mann–Whitney *U* test was used to analyze the comparison of median values by groups, whereas the chi‐squared test was used to compare rates. The analysis results were expressed in medians (minimum–maximum) for continuous variables and in numbers and percentages for categorical variables. Multiple logistic regression analyses (enter method) were used to identify the risk factors associated with irregular menstrual cycles. Age, marital status, and BMI were considered covariates for the analysis, and the results are presented as adjusted odds ratio (AOR) and 95% confidence interval.

The discriminative power (performance) of the IES‐R and PTSD scales for menstrual cycle patterns was assessed upon receiver operating characteristic (ROC) analysis. ROC analysis is used to understand the classification performance of a model through an assessment of its sensitivity and specificity. In particular, ROC analysis helps to investigate the true positive and negative rates of a test/measure. The area under the curve (AUC) is an important indicator of the ROC curve. AUC takes a value between 0 and 1. An AUC value < 0.5 indicates that the test lacks differentiating power. AUC also allows a comparison of the performance of multiple tests (31). In the present study, the IES‐R and PTSD scales were compared. The significance level was taken as *p* < .05. This study was prepared in compliance with the Strengthening the Reporting of Observational Studies in Epidemiology statement.

## 4 RESULTS

The study included 308 women aged between 18 and 49 years. The average age and BMI of the participants were 27.69 and 24.05, respectively. About 50% of the participants held degrees from a university or higher institution, 42.5% were married, and 49% had a monthly income equivalent to their expenses. Among the participants, 42.5% were married, and 41.6% had a pregnancy history. Additionally, 28.9% of participants used family planning methods, 37.9% of these participants preferred the withdrawal method, 23% used condoms, and 16% preferred the pill and tubal ligation.

The data collection was commenced about 9 months after the 2023 Kahramanmaraş earthquake sequence. The prevalence of menstrual cycle irregularity in women aged 18−49 years during the post‐earthquake period was 44.8%. About 14.6% of women reported oligomenorrhea, 1% reported amenorrhea, 12.3% reported menorrhagia, 10.7% reported polymenorrhea, and 10.7% reported metrorrhagia. There was a significant increase in the post‐earthquake prevalence of menstrual cycle irregularity compared to the levels before the earthquake (14.3% vs. 44.8%, *p* < .001; Table 1). Similarly, there was a statistically significant increase in the intensity of menstrual bleeding (*p* < .001). The post‐earthquake changes in menstrual cycle length and duration of bleeding compared to the levels before the earthquake were not statistically significant (*p* > .05).

**TABLE 1:** Comparison of menstrual cycle characteristics of participants before and after the earthquake.

	Before the disaster	After the disaster	Test value	*p* Value
Menstrual cycle length, days, median (min–max)	28 (15–61)	28 (10–90)	49510.5^a^	.342
Duration of bleeding, days, median (min–max)	6 (3–10)	6 (2.12)	46971.5^a^	.830
Menstrual bleeding intensity, *n* (%)				
Light	33 (10.7)^c^	53 (17.2)^d^		
Middle	238 (77.3)^c^	194 (63.0)^d^	15.010^b^	**<.001**
Severe	37 (12.0)^c^	61 (19.8)^d^		
Regular menstrual cycle, *n* (%)				
Yes	264 (85.7)	170 (55.2)	14.893^b^	**<.001**
No	44 (14.3)	138 (44.8)		

*Note*: Values are presented as *n* (%), median (minimum–maximum), Bold denotes statistical significance at *p* < .001

^a^ Mann–Whitney *U* test.

^b^ Chi‐squared test.

^c^ ^,d^The difference in menstrual bleeding intensity.

There was a significant difference between the two groups of women with regular and irregular menstrual cycles after the earthquake in terms of the history of chronic diseases (*p* = .018) and drug use (*p* = .025). Similarly, there was also a statistically significant difference between the groups by smoking status (*p* = .027) and family planning method use (*p* = .021). There was no significant difference in other descriptive characteristics between the two groups of women (*p* > .05) (Table 2). There was no significant intergroup difference by earthquake process characteristics (*p* > .05) (Table 3).

**TABLE 2:** Comparison of participant characteristics between groups.

	Menstrual cycles				
	Regular (*n* = 142)	İrregular (*n* = 166)	Total (*n* = 308)	Test value	*p* Value
Age (years)	24 (18–49)	25 (18–49)	24 (18–49)	10383.5^a^	.082
Body mass index (kg/m^2^)	22.66 (16.80–36.51)	23.41 (16.18–40.06)	23.11 (16.18–40.06)	10642^a^	.162
Age of menarche (years)	13 (9–18)	13 (9–18)	13 (9–18)	10546.5^a^	.120
Education					
Primary secondary school	33 (23.2)	44 (26.5)	77 (25)		
High school	37 (26.1)	40 (24.1)	77 (25)	0.578^b^	.749
University and above	72 (50.7)	82 (49.4)	154 (50)		
Working status					
Not working	135 (79.4)	110 (79.7)	245 (79.5)	0.004^b^	.949
Working	35 (20.6)	28 (20.3)	63 (20.5)		
Marital status					
Single	106 (62.4)	71 (51.4)	177 (57.5)	3.705^b^	.054
Married	64 (37.6)	67 (48.6)	131 (42.5)		
Perceived economic status					
Poor	62 (36.5)	58 (42)	120 (39)		
Medium	85 (50)	66 (47.8)	151 (49)	1.404^b^	.496
Good	23 (13.5)	14 (10.1)	37 (12)		
Chronic illness					
No	150 (88.2)	107 (77.5)	257 (83.4)	5.560^b^	**.018**
Yes	20 (11.8)	31 (22.5)	51 (16.6)		
Drug use					
No	154 (90.6)	113 (81.9)	267 (86.7)	4.275^b^	**.025**
Yes	16 (9.4)	25 (18.1)	41 (13.3)		
Smoking					
No	152 (89.4)	110 (79.7)	262 (85.1)	4.905^b^	**.027**
Yes	18 (10.6)	28 (20.3)	46 (14.9)		
Weekly physical activity					
No	133 (78.2)	108 (78.3)	241 (78.2)	0.000^b^	.996
Yes	37 (21.8)	30 (21.7)	67 (21.8)		
Sleep duration (h/day)	7 (4–15)	7 (4–10)	7 (4–15)	1134.5^a^	.432
Family planning method use					
No	130 (76.5)	89 (64.5)	219 (71.1)	5.319^b^	**.021**
Yes	40 (23.5)	49 (35.5)	89 (28.9)		

*Note*: Values are presented as *n* (%), median (minimum–maximum), Bold denotes statistical significance at *p* < .05

^a^ Mann–Whitney *U* test.

^b^ Chi‐squared test.

**TABLE 3:** Comparison of earthquake‐related features between groups.

	Menstrual cycles				
	Regular (*n* = 170)	İrregular (*n* = 138)	Total (*n* = 308)	Test value^a^	*p* Value
Place of residence during the earthquake					
Village	47 (27.6)	33 (23.9)	80 (26)		
District	74 (43.5)	62 (44.9)	136 (44.2)	0.582	.748
Province	49 (28.8)	43 (31.2)	92 (29.9)		
Where to live after the earthquake					
Village	46 (27.1)	35 (25.3)	81 (26.3)		
District	64 (37.6)	51 (37.0)	115 (37.3)	0.212	.899
Province	60 (35.3)	52 (37.7)	112 (36.4)		
Financial loss due to earthquake					
Slightly damaged–no damage	68 (40.0)	52 (37.7)	109 (35.4)		
Moderately damaged	46 (27.1)	33 (23.9)	79 (25.6)	1.042	.594
Loss of home or workplace	56 (32.9)	53 (38.4)	120 (39)		
Loss of health due to earthquake					
No	92 (64.8)	72 (52.2)	180 (58.4)		
Loss of first degree relative	10 (5.9)	12 (8.7)	22 (7.1)	4.326	.228
Loss of second degree relative	44 (25.9)	44 (31.9)	88 (28.6)		
Being crushed/injured	8 (4.7)	10 (7.2)	18 (5.8)		
Staying in a tent/container after the earthquake					
No	104 (61.2)	75 (54.3)	179 (58.1)	1.459	.227
Yes	66 (38.8)	63 (45.7)	129 (41.9)		

*Note*: Values are presented as *n* (%).

^a^Chi‐squared test.

The PTSD scale cut‐off point was taken as 24. Accordingly, the prevalence of PTSD is 22.7%. IES‐R score was 43.65 ± 16.21 (2−88). A comparison of the PTSD and IES‐R scales on the study groups is shown in Table 4. For the group with menstrual irregularities, the median scores of the IES‐R scale total score (*p* = .002) and the sub‐domains of intrusion (*p* = .004) and hyperarousal (*p* = .004) were significantly higher. Similarly, the median value of the PTSD scale was significantly higher in the group with menstrual irregularities (*p* = .001) (Table 3).

**TABLE 4:** Comparison of participants' Impact of Events Scale and Post‐traumatic Stress Scale scores between groups.

	Menstrual cycles				
	Regular (*n* = 170)	İrregular (*n* = 138)	Total (*n* = 308)	Test value	*p* Value
IES‐R					
İntrusion	16 (1–30)	18 (1–32)	17 (1–32)	9501^a^	**.004**
Avoidance	14 (4–25)	15 (0–32)	14 (0–32)	10240^a^	.055
Hyperarousal	11.5 (1–24)	14 (0–24)	12 (0–24)	9524^a^	**.004**
IES‐R total	40 (8–71)	46 (2–88)	44 (2–88)	9340^a^	**.002**
PTSD	16 (0–33)	19.5 (0–36)	17 (0–36)	9175^a^	**.001**

*Note*: Values are presented as *n* (%), median (minimum–maximum), Bold denotes statistical significance at p < .05.

^a^Mann–Whitney *U* test.

Risk factors for menstrual cycle irregularity were tested using the logistic regression analysis. Upon adjustment for age, BMI, and marital status, comorbid chronic diseases (AOR = 2.027, *p* = .038) and smoking (AOR = 2.174, *p* = .024) were the risk factors for menstrual cycle irregularity in Model 1a. Both the IES‐R scale score (AOR = 1.019, *p* = .017) and PTSD scale score (AOR = 1.046, *p* = .012) were the risk factors for menstrual cycle irregularity for Models 1a and 3a (Table 5).

**TABLE 5:** Determinants of menstrual cycle irregularity.

	Model 1a AOR (%95 CI)	*p*	Model 2b OR (%95 CI)	*p*	Model 3a AOR (%95 CI)	*p*	Model 4b OR (%95 CI)	*p*
Chronic illness	2.027 (1.038–3.958)	**.038**	1.975 (1.053–3.704)	**.034**	2.104 (1.075–4.117)	**.030**	2.060 (1.099–3.863)	**.024**
Smoking	2.174 (1.108–4.264)	**.024**	2.035 (1.056–3.922)	**.034**	2.199 (1.118–4.327)	**.022**	2.063 (1.068–3.988)	**.031**
Scale	1.019 (1.003–1.035)	**.017**	1.020 (1.005–1.035)	**.009**	1.046 (1.013–1.080)	**.012**	1.048 (1.015–1.081)	**.004**

^a^Adjusted for age, marital status, and BMI

^b^No adjustments were made.

*Note*: Bold denotes statistical significance at *p* < .05,. Scale: for Models 1a and 2b, the scale represents IES‐R, for Models 3a and 4b, the scale represents PTSD.

Abbreviations: AOR, adjusted odds ratio; OR, odds ratio; CI, confidence interval.

**FIGURE 1:**
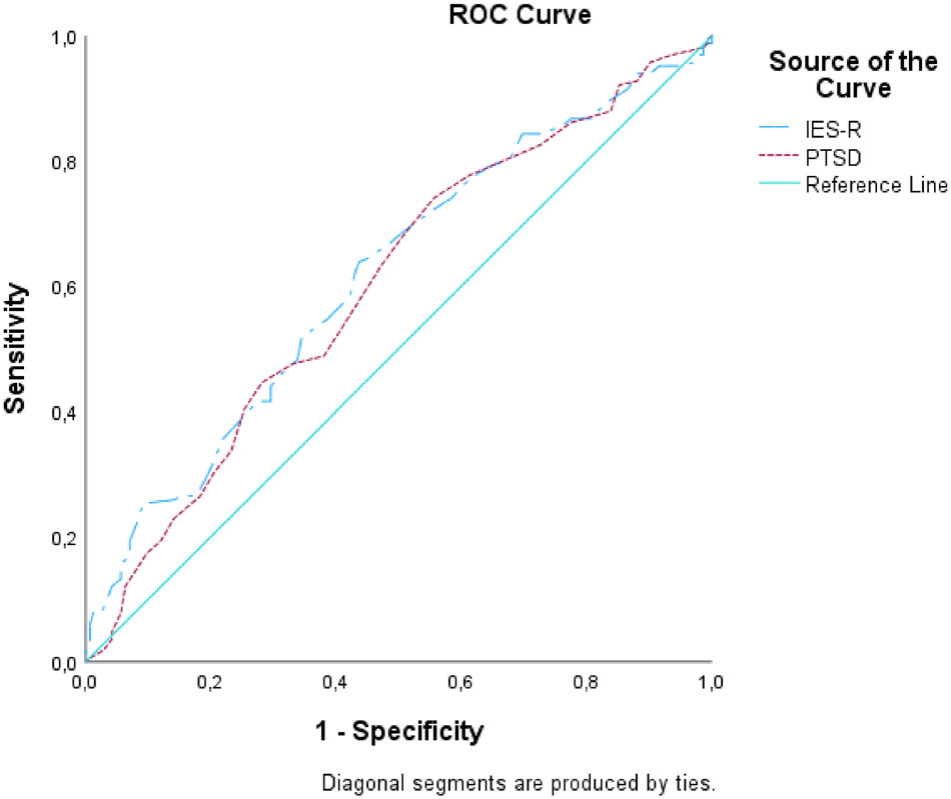
ROC analysis plot for IES‐R and PTSD scale.

The ROC analysis, used in the present study to investigate the effectiveness of the IES‐R and PTSD scales in determining the regularity and irregularity status of the menstrual cycle, served as a secondary analysis (Table 6, Figure 1). Based on the ROC analysis on the IES‐R and PTSD scales, an individual selected from the group with irregular menstruation had a 64.12% (IES‐R) and 61.76% (PTSD) higher threshold test score compared to an individual selected from the group with regular menstruation. This was a statistically significant value. For a cut‐off value of 45.50 with IES‐R, the sensitivity and specificity values were 64.12% and 53.62%, respectively, whereas, for a cut‐off value of 18.5 with PTSD, the sensitivity and specificity values were 61.76% and 50.72%, respectively. When the cut‐off score of the IES‐R scale was set at 45.50, it had higher sensitivity in detecting irregular menstrual cycles (sensitivity: 64.12% [56.42−71.32]). Other descriptive results are shown in Table 6 and Figure 1.

**TABLE 6:** ROC analysis results.

	IES‐R	PTSD
AUC (%95 CI)	0.602 (0.538–0.666)*	0.609 (0.546–0.672)*
Cutoff	≥45.50	≥18.5
Sensitivity	64.12 (56.42–71.32)	61.76 (54.01–69.10)
Specificity	53.62 (44.94–62.15)	50.72 (42.09–59.33)
PPV	63.01 (57.95–67.79)	60.69 (55.67–65.50)
NPV	54.81 (48.48–60.99)	51.85 (45.56–58.08)
Accuracy	59.42 (53.70–64.95)	56.82 (51.08–62.42)

*denotes statistical significance at *p* < .05

Abbreviations: PPV, positive predictive value; NPV, negative predictive value.

## 5 DISCUSSION

Traumatic events such as earthquakes can have an impact on general health status but are especially considered significant stressors vis‐à‐vis the reproductive health of women. Such major traumas may be associated with increased stress levels, disrupted hormone balance, and irregular menstrual cycles (Liu et al., 2010). This study aimed to investigate the relationship between alterations in menstrual cycles and PTS in earthquake‐affected women. As a result of the study, there was a marked increase in menstrual cycle irregularities among women. There were significant differences between women with regular and irregular menstrual cycles by PTS symptoms, comorbid chronic diseases, prolonged medication use, smoking, and family planning method use. It was said that comorbid chronic diseases, smoking, and elevated IES‐R and PTSD scale scores increased the risk of menstrual irregularities.

Previous studies reported that 14%−26.7% of women of reproductive age had irregular menstrual cycles (Farhan et al., 2020; Whitaker & Critchley, 2016). The present study was conducted 9 months post‐earthquake and indicated that 44.8% of the surviving women had menstrual cycle irregularities, a significant increase compared to the pre‐earthquake period. Upon a literature review, the post‐earthquake prevalence of menstrual irregularity was reported as 21% following the Wenchuan earthquake in China (Li et al., 2011) and 42.4% following the 2017 earthquake in Kermanshah, Iran (Rajabi et al., 2022). Similarly, a study with female students after the Wenchuan earthquake in China reported the rate of abnormal menstruation as 76.6% (Liu et al., 2010). Major stressors, such as earthquakes, significantly increase the prevalence of irregular menstrual cycles in women of reproductive age compared to the general population. Stress and traumatic events may affect the menstrual cycle by influencing hormone production through the hypothalamus–pituitary–adrenal axis (Hantsoo et al., 2023).

Exposure to earthquakes and witnessing the loss of relatives and injuries may increase the risk of developing PTSD (35). The prevalence of PTSD in study participants was 22.7%. Previous studies reported that PTSD was frequent in earthquake survivors, with a prevalence varying between 19% and 51% (Dai et al., 2016; İlhan et al., 2023; Thapa & Acharya, 2017; Wu et al., 2014; Xie et al., 2017). Consistent with the relevant literature, symptoms of PTSD in the present study were indicative of an increased risk of mental health problems in post‐earthquake women. In this study, there was a significant difference in PTSD symptoms between women with regular and irregular menstrual cycles. Women with PTS symptoms were 1.02 (IES‐R) and 1.04 (PTSD) times more likely to report irregular menstrual cycles. This difference remained significant even upon adjustment for covariates, including age, marital status, and BMI. Liu and Yang (Farooqui et al., 2017) reported in their study that irregular menstrual cycles were significantly higher in students with positive PTSD symptoms compared to students without symptoms. Another study, which was performed 9 months after the Great East Japan Earthquake, reported that PTSD was associated with the severity of dysmenorrhea in high school students (Takeda et al., 2013b) with premenstrual syndrome (Takeda et al., 2013a).While a small amount of stress can help people adapt to changes and overcome difficulties, too much stress can have detrimental effects on an individual's physical and mental health (O'Connor et al., 2021). Earthquake‐related trauma and stress can affect the nervous and endocrine system, leading to increased serum cortisol levels. This may disrupt hormonal balance by acting on the hypothalamus–pituitary–ovarian axis (Raise‐Abdullahi et al., 2023) and induce irregular menstrual cycles (Hantsoo et al., 2023).

Menstrual irregularities may be affected by lifestyle‐associated factors, including physical activity, sleep, environmental factors, and chronic diseases (Attia et al., 2023). In the present study, there was a significant difference between women with regular and irregular menstrual cycles by comorbid chronic diseases and medication use. Women with chronic diseases were approximately twice as likely to report irregular menstrual cycles. Regular insulin, thyroid, ovarian, adrenal, and hypothalamic hormone production are required for a normal menstrual cycle. This cycle is regulated by neuroendocrine mechanisms (Berga & Naftolin, 2012). Chronic diseases, including diabetes and thyroid disease, can affect the menstrual cycle and induce irregularities. Furthermore, the drugs used may induce alterations in the menstrual cycle by affecting hormone levels, neurotransmitters, and liver enzymes (Greenwell et al., 2023). Smoking is considered a modifiable risk factor associated with irregular menstruation (Attia et al., 2023). In the present study, there was a significant difference between women with regular and irregular menstrual cycles by smoking status. Women smokers were approximately twice as likely to report irregular menstrual cycles. Consistent with the results of previous studies, this suggested that smoking was associated with irregular menstrual cycles (Kwak et al., 2019; Zafar, 2020). Family planning methods, especially hormonal contraceptives, play an important role in regulating the menstrual cycle. These methods suppress ovulation and regulate the cycle by regulating estrogen and progesterone levels (Bradley & Gueye, 2016). Nevertheless, hormonal contraceptives can also cause irregularities by altering the body's natural hormone cycle (De Leo, Musacchio, Cappelli, Piomboni, & Morgante, 2016). In the present study, there were significant differences between women with regular and irregular menstrual cycles using a family planning method. This result was consistent with the reports of previous studies (Mohammed & Abdel‐Aleem, 2017; Uçar et al., 2015). This may be explained by hormonal imbalances and menstrual irregularities caused by discontinuation or irregular use of hormonal contraceptives and by the fact that some intrauterine devices can cause irregular menstrual cycles. These results are important for better understanding various interactions in the menstrual cycle and assessing the potential health impacts of these factors. Identifying groups at risk of deteriorated reproductive health in such situations as earthquakes can help organizations provide more effective assistance in the aftermath of such events.

For irregular menstrual cycles, the AUC was approximately the same for IES‐R and PTSD. Sensitivity is important during the post‐earthquake period to refer to as many cases as possible for a more comprehensive diagnostic examination (Baldessarini et al., 1983). The sensitivity of IES‐R (64.12) was higher compared to that of PTSD (61.76). The fact that PTSD is more related to PTSD and shows its effects in the long term may account for this difference. In the short term, after the earthquake, the IES‐R scale may be considered a practical scale to predict irregular menstrual cycles. The cut‐off point for menstrual cycle irregularity was set to 45.50. Women who scored above 45.50 on the IES‐R scale should be monitored more closely.

## 6 CONCLUSION AND RECOMMENDATIONS

The study found that 44.8% of the participants experienced menstrual cycle irregularities after the 2023 Kahramanmaraş earthquake sequence in Turkey. PTS symptoms, chronic diseases, medication use, smoking, and family planning method are associated with irregular menstrual cycles. The prevalence of PTSD was 22.7%, and this was associated with women who experienced menstrual irregularities. Therefore, post‐earthquake mental health recovery programs must be planned and implemented to specifically include women with menstrual irregularities. This study evaluated earthquake‐affected individuals, but no comparison was made with control groups. Future studies can make a more comprehensive comparison with control groups from regions not affected by the earthquake. At the same time, considering that psychosocial effects are important in the menstrual irregularities experienced by women after the earthquake, it is recommended that more research be conducted in this area.

## AUTHOR CONTRIBUTIONS

**Sibel Kiyak**: Conceptualization; methodology; software; data curation; formal analysis; investigation; writing—original draft; writing—review and editing; project administration; visualization. **Serap Batı**: Conceptualization; methodology; supervision; writing—review and editing; investigation.

## FUNDING INFORMATION

Open access funding provided by the Scientific and Technological Research Council of Türkiye (TÜBİTAK).

### PEER REVIEW

The peer review history for this article is available at https://publons.com/publon/10.1002/brb3.70034.

## Data Availability

The data that support the findings of this study are available from the corresponding author upon reasonable request.
